# Removal of Minnie-Ball from Pelvis

**Published:** 1874-04

**Authors:** 


*Removal of Minnie-Ball from Pelvis.*

Dr. A. W. Calhoun, of Atlanta, Ga., in the presence of the editors of this journal, succeeded in removing a minnie-ball from the pelvis of an ex-Confederate officer to-day, the 21st inst., which that unfortunate gentleman had been carrying for over ten years, with great drainage to the system and intense suffering. The operation was performed with great skill. Recovering perfectly from the shock of the operation and the chloroform, the patient turned easily in his bed from side to side, with no hemorrhage and circulation normal; still the condition of the anterior walls of the rectum and the muscles composing the floor of the pelvis, which was revealed by the operation, excited some apprehension of an unfavorable termination. If he recovers we will rejoice. If he does not, while we will have nothing to regret in the operation—as it was certainly most skillfully performed—we could not be greatly surprised, learning the exterior disintegration of the parts surrounding the location of the ball. We trust the case will be reported in full in the future. T. S. P.

